# Posterior Bulboprostatic Excision and Primary Anastomosis for Pelvic Fracture Urethral Injury: Long-term Objective and Patient-reported Outcomes

**DOI:** 10.1590/S1677-5538.IBJU.2025.0509

**Published:** 2025-10-30

**Authors:** Jakob Klemm, Max C. Wagner, Robert J. Schulz, Navid Roessler, Margit Fisch, Roland Dahlem, Malte W. Vetterlein

**Affiliations:** 1 University Medical Center Hamburg-Eppendorf Department of Urology Hamburg Germany Department of Urology, University Medical Center Hamburg-Eppendorf, Hamburg, Germany

**Keywords:** Urethral Stricture, Anastomosis, Surgical, Treatment Outcome

## Abstract

**Purpose:**

Posterior bulboprostatic excision and primary anastomosis (EPA) is considered standard of care for obliterative or disruptive pelvic fracture urethral injuries (PFUIs), yet validated patient-reported outcomes (PROMs) in this setting remain limited. We aimed to evaluate long-term reintervention-free survival (RFS) and PROMs following EPA.

**Patients and Methods:**

This retrospective study included male patients undergoing transperineal bulboprostatic EPA for PFUI between 2014 and 2024 at a tertiary reconstructive referral center. Data collected included trauma etiology, comorbidities, prior interventions, operative details, and follow-up duration. Co-primary endpoints were RFS estimated by Kaplan-Meier analysis, and PROMs assessed using validated instruments.

**Results:**

Seventy patients (median age 48 years) underwent EPA. Initial management included suprapubic catheter (77%), endoscopic (21%), or open realignment (1.4%). Median operative time was 77 minutes; median follow-up was 53 months. RFS was 87% at 2 years and 84% at 5 years. PROMs—available in 53% of patients at median 71 months—included moderate voiding/incontinence symptoms (median LUTS score 6; ICIQ-UI SF 7), severe erectile dysfunction (IIEF-EF 7), preserved ejaculatory function (MSHQ-Ej 24), high satisfaction (ICIQ-S 21; global satisfaction 9), and negligible decision regret (median 0). Limitations include retrospective design and incomplete PROM data (53% response rate).

**Conclusions:**

Bulboprostatic EPA offers durable anatomical success and high long-term patient satisfaction despite persistent functional impairments largely linked to initial trauma. Most patients expressed minimal regret and willingness to repeat the procedure. These outcomes reinforce EPA's role as the standard of care in PFUI management.

## INTRODUCTION

Bulboprostatic excision and primary anastomosis (EPA) is the gold standard for managing pelvic fracture urethral injuries (PFUIs) involving complete urethral disruption. These injuries typically result from road traffic accidents, motor vehicle collisions, or falls from height and most often affect otherwise healthy men in midlife who suddenly face profound functional and quality-of-life impairments. In cases of partial urethral rupture, primary realignment may be feasible and is associated with a reduced risk of stricture formation. In contrast, complete ruptures generally require urinary diversion followed by delayed urethroplasty (1).

Although bulboprostatic EPA for PFUI is widely performed and strongly endorsed by both American (2) and European guidelines (1)—with numerous surgical series available—there remains a notable lack of data on patient-reported outcome measures (PROMs). Most existing studies focus exclusively on anatomical or functional endpoints, often overlooking quality-of-life domains that are highly relevant to this patient population (3–19). This is particularly striking given that current urethral stricture disease guidelines explicitly recommend the use of PROMs to assess patient satisfaction and outcomes (20).

This gap is especially important because PFUIs predominantly affect young men in the prime of life. For these individuals, treatment goals extend well beyond technical success—they include the restoration of continence, sexual function, and overall well-being after a life-altering trauma. In this context, PROMs are essential for capturing outcomes that truly matter to patients.

We hypothesized that patients undergoing bulboprostatic EPA for PFUI would report high levels of treatment satisfaction but may experience long-term functional sequelae, particularly affecting urinary continence and sexual function. To address this knowledge gap, we analyzed long-term functional and patient-reported outcomes in a contemporary cohort of patients who underwent bulboprostatic EPA for PFUI at our high-volume reconstructive referral center over the past decade.

## PATIENTS AND METHODS

### Study Population and Data Extraction

This retrospective observational study was approved by the Ethics Committee of the Medical Council of Hamburg (No. PV4123) and conducted in accordance with the Hamburg Hospital Act (§12.1 HmbKHG). We identified all male patients who underwent bulboprostatic EPA, defined by the operation and procedure classification system (OPS) code 5-584.5, between June 2014 and May 2024. Eligible patients had a documented history of PFUI with partial or complete urethral disruption at the bulbomembranous junction. Patients with posterior urethral stenoses of other etiologies, such as vesicourethral anastomotic stenosis following radical prostatectomy, were excluded. Electronic medical records were reviewed to extract data on demographics, trauma characteristics, stricture extent, prior interventions, and surgical details. Follow-up was conducted via structured telephone interviews and an online questionnaire.

### Study End Points

Endpoints included both objective and subjective outcomes. Objective outcomes comprised functional success, defined as reintervention-free survival, with recurrence indicated by any postoperative intervention for recurrent urethral stricture (21) and perioperative complications within 30 days, classified according to the Clavien–Dindo system (22).

Subjective outcomes were assessed using a comprehensive set of validated PROMs. All instruments use linear scoring systems and have been validated to assess patient-centered outcomes across key domains, including voiding symptoms, continence, erectile and ejaculatory functions, treatment satisfaction, and decision regret. Lower urinary tract symptoms (LUTS) were evaluated using the Urethral Stricture Surgery (USS) PROM six-item LUTS score ranging from 0 to 24 (23, 24); higher scores indicate more severe symptoms. Urinary incontinence was assessed using the International Consultation on Incontinence Questionnaire – Urinary Incontinence Short Form (ICIQ-UI SF), comprising three items and yielding a total score between 0 and 21 (25) with higher scores reflecting greater incontinence severity. Erectile function was measured using the erectile function domain of the International Index of Erectile Function (IIEF-EF), which includes six items and produces a score ranging from 1 to 30 (26); higher scores indicate better erectile function. To account for non-intercourse responses, scoring was adjusted according to the method proposed by Vickers et al. (27). Ejaculatory function was assessed using the ejaculatory function domain of the Male Sexual Health Questionnaire (MSHQ-Ej), which includes seven items and yields a score from 1 to 35 (28) with higher scores indicating better function. Satisfaction with surgical outcomes was measured using the ICIQ-Satisfaction module (ICIQ-S), consisting of six items forming an outcome score between 0 and 24, along with a separate item for overall satisfaction with surgery rated on a scale from 0 to 10; (29) higher scores reflect greater satisfaction. Decisional regret was evaluated using the five-item Decision Regret Scale (DRS), with a total score ranging from 0 to 100 (30); higher scores indicate greater regret regarding the decision to undergo surgery.

### Perioperative Management and Surgical Procedure

Preoperative evaluation followed our institutional protocol and included medical history, physical examination, urinalysis, and combined retrograde urethrography with voiding cystourethrography to assess stenosis extent. All patients had a suprapubic catheter in place before surgery. Procedures were performed by two experienced reconstructive urologists (MF, RD) using a standardized perineal approach, as originally described by Webster (3, 31).

Briefly, the patient was positioned in lithotomy, and a midline perineal incision was made. The bulbospongiosus muscle was dissected from the corpus spongiosum, and the bulbar urethra was mobilized to the pelvic floor. A 22 F metal sound was introduced through the external meatus to identify the distal edge of the stenosis, which was then transected and spatulated just distal to the fibrotic cone. Proximal dissection continued until healthy urethra at the prostatic apex was reached and similarly spatulated. A tension-free end-to-end anastomosis was performed using eight interrupted 4-0 absorbable monofilament sutures. Ancillary maneuvers—such as extensive urethral mobilization, corporal body separation, or inferior pubectomy—were used when needed to bridge the urethral gap (3, 31, 32). A 16 F silicone catheter was placed transurethrally, and a drain was positioned between the bulbar urethra and bulbospongiosus muscle, typically removed after 24–48 hours. Patients were usually discharged on postoperative day 5. At three weeks postoperatively, a voiding cystourethrogram was performed. In the absence of contrast extravasation and with successful spontaneous voiding, the suprapubic catheter was removed. If extravasation was present, the catheter was maintained for one additional week, followed by repeat imaging. Both the surgical technique and postoperative management were standardized across the cohort.

## Statistical Analysis

Baseline clinical characteristics were summarized descriptively. Continuous variables are presented as medians with interquartile ranges (IQRs) and as means with standard deviations (SDs); categorical variables are shown as absolute frequencies and percentages. Median follow-up among censored patients was estimated using the reverse Kaplan–Meier method. Reintervention-free survival was analyzed and visualized with Kaplan–Meier survival curves. To retrospectively assess recalled erectile function after the initial trauma but prior to bulboprostatic EPA, patients were asked: "Did you notice any deterioration in your erectile function after the traumatic urethral injury/pelvic trauma?" Response options were: 1 – Yes, significantly worse; 2 – Yes, somewhat worse; 3 – No, unchanged; 4 – No, somewhat improved; 5 – No, significantly improved. Validated PROMs were assessed according to their respective scoring guidelines. Scores are presented as medians with IQRs and were visualized using violin plots. All statistical analyses were performed using Stata, Release 18 (StataCorp LLC, College Station, TX, USA).

## RESULTS

### Clinical Baseline Characteristics

A total of 70 patients underwent bulboprostatic EPA between June 2014 and May 2024 at our institution. Baseline characteristics are summarized in Table-1. The median age at surgery was 48 years (IQR 31–56), and the median body mass index (BMI) was 26 kg/m^2^ (IQR 24–28). Concomitant bladder neck injury was present in 7 patients (10%), and rectal injury occurred in 6 patients (8.8%) at the time of initial trauma. Initial urethral management consisted of suprapubic catheter placement in 54 patients (77%), endoscopic realignment in 15 patients (21%), and open realignment in 1 patient (1.4%). The median interval from trauma to reanastomosis was 11 months (IQR 6–20), and the median operative time of bulboprostatic EPA was 77 minutes (IQR 65–93). To achieve a tension-free anastomosis, corporal splitting was performed in 65 patients (93%), and inferior pubectomy was required in 2 cases (2.9%).

**Table 1:** Clinical baseline and surgical characteristics in 70 men undergoing transperineal bulboprostatic excision and primary anastomosis between June 2014 and May 2024 at a tertiary reconstructive referral center.

Baseline and surgical characteristics		
Patients, n (%)		70 (100)
Age at surgery (yr), median (IQR); mean (SD); range		48 (31–56); 44 (15); 16–72
BMI, median (IQR); mean (SD); range		26 (24–28); 26 (4.2); 18–36
**Comorbidities, n (%)**		
	Diabetes	2 (2.9)
	Hypertension	9 (13)
	Smoking	26 (37)
**ASA physical status, n (%)**		
	I	12 (17)
	II	48 (69)
	III	10 (14)
**Concomitant primary trauma characteristics, n (%)**		
	Rectal injury	6 (8.8)
	Bladder neck injury	7 (10)
**Initial urethral management, n (%)**		
	Suprapubic catheter only	54 (77%)
	Endoscopic realignment	15 (21)
	Open realignment	1 (1.4)
	Time from initial trauma to reanastomosis (months), median (IQR); mean (SD); range	11 (6-20); 36 (86); 1–550
	Operative time (minutes), median (IQR); mean (SD); range	77 (65–93); 79 (47); 44–170
**Ancillary maneuvers performed intraoperatively, n (%)**		
	Corporal splitting	65 (93%)
	Inferior pubectomy	2 (2.9%)

ASA = American Society of Anesthesiologists; BMI = body mass index (kg/m²); IQR = interquartile range; SD = standard deviation.

### Reintervention-Free Survival and Postoperative Complications

**Figure 1:**
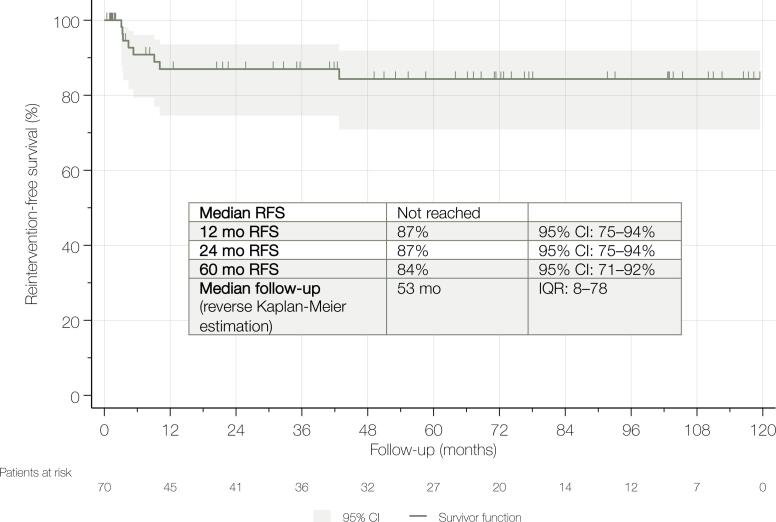
Kaplan-Meier curve depicting reintervention-free survival in 70 patients undergoing transperineal bulboprostatic excision and primary anastomosis for pelvic fracture urethral injury.

At a median follow-up of 53 months (IQR 8–78), 8 patients (11%) required reintervention for recurrent urethral stricture. The estimated reintervention-free survival was 87% at 2 years and 84% at 5 years (Figure-1). Specifically, five patients underwent endoscopic interventions, including internal urethrotomy (n = 5); in one case, this was combined with transurethral scar tissue resection. Three patients required repeat bulboprostatic EPA due to recurrent stricture. Of these, one ultimately underwent permanent suprapubic catheter placement following failed revision surgery. Two patients (2.9%) experienced major postoperative complications classified as Clavien–Dindo grade ≥IIIa. Both presented with wound infections and localized abscess formation, which were managed with drainage under local anesthesia.

CI = confidence interval; IQR = interquartile range; RFS = reintervention-free survival.

### Patient-reported Outcome Measures

**Figure 2:**
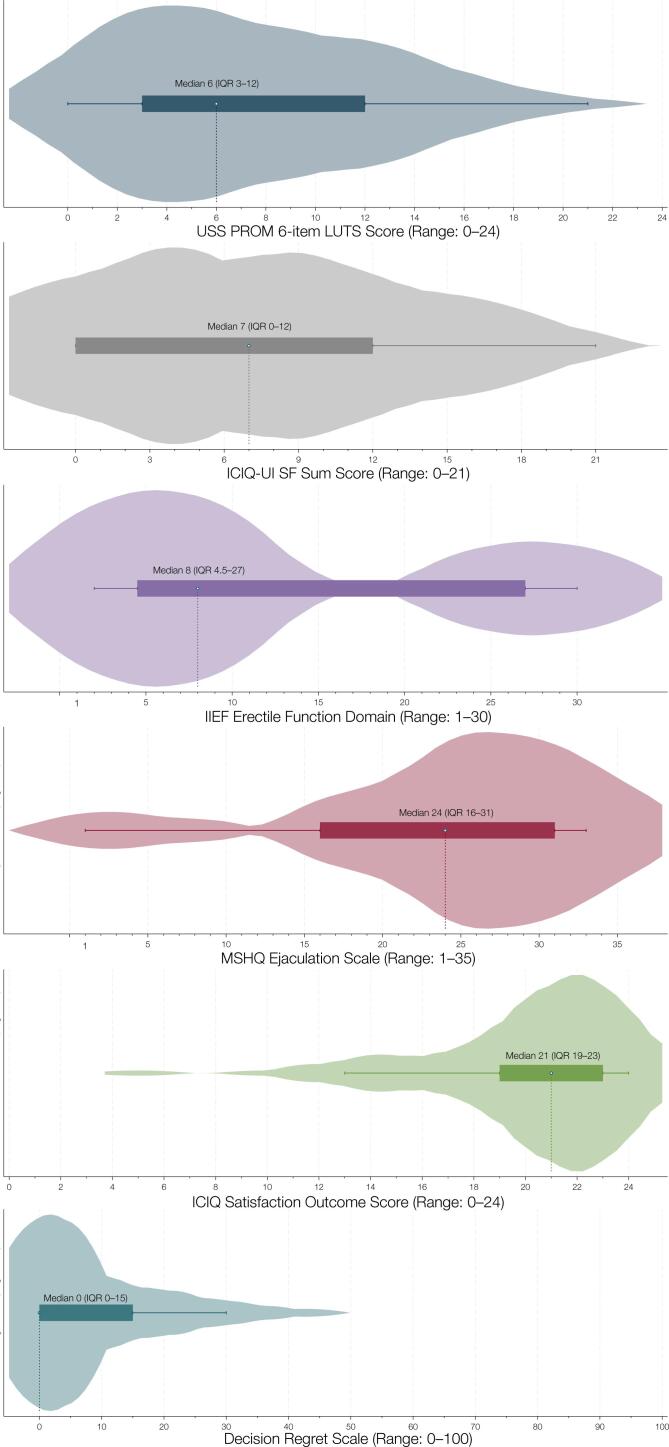
Violin plots illustrating the distribution of scores for validated patient-reported outcome measures in 37 of 70 patients undergoing bulboprostatic excision and primary anastomosis. ICIQ indicates International Consultation on Incontinence Questionnaire; ICIQ-UI SF, International Consultation on Incontinence Questionnaire–Urinary Incontinence Short Form; IIEF, International Index of Erectile Function; IQR, interquartile range; LUTS, lower urinary tract symptoms; MSHQ, Male Sexual Health Questionnaire; USS PROM, Urethral Stricture Surgery Patient-Reported Outcome Measure.

**Figure 3:**
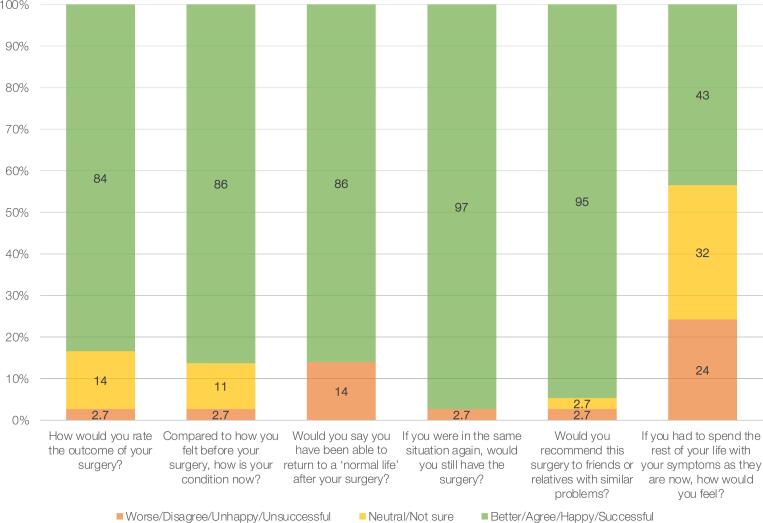
ICIQ-Satisfaction (ICIQ-S) outcomes questions survey results (n = 37). Percentages may not add up to 100%, as they are rounded.

PROMs were collected at a median follow-up of 71 months (IQR 49–103), with complete data available for 37 patients (53%). Of all patients who responded to the retrospective question on erectile function after the initial trauma but prior to urethral reconstruction, 29 (78%) reported that their erectile function had become significantly or somewhat worse compared to their pre-trauma baseline. The distribution of the validated postoperative PROM scores is illustrated in Figure-2. The median postoperative LUTS score was 6 (IQR 3–12), indicating generally restored voiding function. The median ICIQ-UI SF score was 7 (IQR 0–12), corresponding to moderate urinary incontinence.(33) Erectile function, as measured by the IIEF-EF domain, had a median score of 8 (IQR 4.5–27), suggesting substantial variability in postoperative outcomes. Notably, the distribution was bimodal, with two distinct peaks indicating subgroups with preserved versus impaired erectile function. Median ejaculatory function, assessed via the MSHQ-Ej, was 24 (IQR 16–31), suggesting relatively better preservation of this domain. The median ICIQ-S outcome score was 21 (IQR 19–23), and the median overall satisfaction with surgery was 9 (IQR 6–10), reflecting a high level of patient satisfaction. Figure-3 illustrates the distribution of responses to the six individual ICIQ-S items, which collectively form the ICIQ-S outcome score (range: 0–24). Finally, the median DRS score was 0 (IQR 0–15), indicating negligible regret regarding the decision to undergo surgery.

## DISCUSSION

Successful treatment of PFUIs through open reconstruction hinges on two central outcomes: long-term urethral patency without the need for reintervention and optimal functional recovery following severe trauma. This includes satisfactory voiding, continence, preservation of sexual function, high treatment satisfaction, and minimal decision regret. While multiple studies have reported on anatomical outcomes and surgical techniques for bulboprostatic EPA in the context of PFUI (3–19), this is the first study to incorporate a comprehensive battery of validated PROMs—offering a detailed view of patient-centered outcomes in this high-impact clinical scenario.

Our findings confirm that bulboprostatic EPA offers durable reintervention-free survival, with 2- and 5-year success rates of 87% and 84%, respectively. These results are consistent with previously reported outcomes and reinforce the status of bulboprostatic EPA as the gold standard for managing complete PFUI (3–19). Importantly, this study goes beyond technical success to examine functional outcomes from the patient's perspective, an aspect that has been underrepresented in literature to date.

Despite restored urethral patency in the majority of cases, our PROM data show that many patients continue to experience moderate voiding symptoms and urinary incontinence. These findings underscore the fact that anatomical success does not necessarily equate to complete functional recovery. While earlier studies have described incontinence following bulboprostatic surgery (3–19), definitions of continence and incontinence vary widely, and few have used validated tools to assess this domain. This study provides the first PROM-based quantification of urinary function after bulboprostatic EPA for PFUI, revealing meaningful residual symptoms that may warrant further management in selected patients.

Sexual function emerged as another domain with notable impairment. Erectile function, as assessed by the IIEF-EF, was the most adversely affected PROM, with scores indicating relatively severe dysfunction in a substantial proportion of patients. Interestingly, the bimodal distribution of IIEF-EF scores suggests heterogeneity in postoperative outcomes—likely reflecting differences in the severity of initial trauma and preexisting erectile dysfunction. In fact, 78% of patients had documented erectile dysfunction prior to surgery, consistent with the understanding that sexual function is often compromised by the injury itself rather than the reconstructive procedure. This aligns with the limited number of studies that have applied validated PROMs in this setting. Two such studies demonstrated that erectile dysfunction was primarily attributable to the initial trauma, with reconstructive surgery having little further impact on sexual outcomes (14, 19). Our findings support this conclusion and emphasize the importance of preoperative counseling regarding realistic expectations for postoperative sexual function. In contrast to erectile dysfunction, ejaculatory function appeared to be relatively well preserved in our cohort. While few prior studies have addressed this specific domain, our results indicate that ejaculatory function may remain intact in many patients—even in the context of extensive urethral reconstruction. Further research is warranted to explore the mechanisms underlying this preservation and to confirm these findings in larger cohorts.

Patient satisfaction and decision-making confidence are critical—yet often overlooked—outcomes in reconstructive urology. To our knowledge, this is the first study to assess both treatment satisfaction and decisional regret using validated instruments in a PFUI population undergoing bulboprostatic EPA. The high satisfaction scores and low DRS values observed in our cohort suggest that, despite ongoing functional limitations, most patients viewed their surgical outcomes positively and would choose the intervention again. This highlights the overall value of bulboprostatic EPA not only as a technically effective procedure but also as a meaningful intervention from the patient's perspective.

Our findings should be interpreted considering several limitations. First, the retrospective design and relatively small sample size limited our ability to perform multivariable analyses to identify predictors of adverse outcomes. Second, the cross-sectional nature of PROM collection may not fully capture longitudinal changes in patient function and satisfaction. Third, the lack of preoperative PROM data restricts our ability to quantify change over time, particularly in functional domains such as continence and sexual health. However, our inclusion of treatment satisfaction and decisional regret offers important complementary insight into the overall patient experience. Fourth, recall and response bias cannot be excluded, particularly in retrospective assessments of preoperative function or satisfaction. Fifth, although the response rate of 53% for the PROMs is suboptimal, this limitation is common in retrospective and survey-based studies. Consequently, the available data may be subject to response bias, as patients who complete PROMs are often more motivated or satisfied than non-responders. Nonetheless, this study fills a literature gap by applying a validated, multi-dimensional PROM framework to a procedure that is both technically demanding and functionally consequential. By systematically evaluating the outcomes that matter most to patients—beyond anatomical success—we offer a more complete understanding of the benefits and limitations of bulboprostatic EPA for PFUI.

## CONCLUSIONS

Bulboprostatic EPA offers durable reintervention-free survival and remains the gold standard for the surgical management of PFUIs. While validated PROMs highlight ongoing functional challenges—particularly related to urinary incontinence and erectile dysfunction—these issues likely reflect the severity of the initial trauma rather than surgical shortcomings. Despite these limitations, patient-reported satisfaction was high, and decisional regret was minimal. Most patients indicated they would choose the procedure again, underscoring the meaningful clinical and quality-of-life benefits of bulboprostatic EPA. These findings emphasize the importance of incorporating PROMs into routine outcome assessment and support the role of bulboprostatic EPA as a patient-centered, effective treatment for PFUI.

## Data Availability

All data generated or analysed during this study are included in this published article
